# The Snark was a Boojum - reloaded

**DOI:** 10.1186/1742-9994-12-S1-S20

**Published:** 2015-08-24

**Authors:** Simone Macrì, S Helene Richter

**Affiliations:** 1 Section of Behavioural Neuroscience, Department of Cell Biology and Neuroscience, Istituto Superiore di Sanità, Viale Regina Elena 299, 00161, Roma, Italy; 2 Department of Behavioural Biology, Institute of Neuro and Behavioural Biology, University of Münster, Badestraße 13, 48149 Münster, Germany

**Keywords:** Translational Neuroscience, Behaviour, Animal Models, Reproducibility, Adaptive Plasticity, Development

## Abstract

In this article, we refer to an original opinion paper written by Prof. Frank Beach in 1950 (“The Snark was a Boojum”). In his manuscript, Beach explicitly criticised the field of comparative psychology because of the disparity between the original understanding of comparativeness and its practical overly specialised implementation. Specialisation encompassed both experimental species (rats accounted for 70% of all subjects) and test paradigms (dominated by conditioning/learning experiments). Herein, we attempt to evaluate the extent to which these considerations apply to current behavioural neuroscience. Such evaluation is particularly interesting in the context of “translational research” that has recently gained growing attention. As a community, we believe that preclinical findings are intended to inform clinical practice at the level of therapies and knowledge advancements. Yet, limited reproducibility of experimental results and failures to translate preclinical research into clinical trial sindicate that these expectations are not entirely fulfilled. Theoretical considerations suggest that, before concluding that a given phenomenon is of relevance to our species, it should be observed in more than a single experimental model (be it an animal strain or species) and tested in more than a single standardized test battery. Yet, current approaches appear limited in terms of variability and overspecialised in terms of operative procedures. Specifically, as in 1950, rodents (mice instead of rats) still constitute the vast majority of animal species investigated. Additionally, the scientific community strives to homogenise experimental test strategies, thereby not only limiting the generalizability of the findings, but also working against the design of innovative approaches. Finally, we discuss the importance of evolutionary-adaptive considerations within the field of laboratory research. Specifically, resting upon empirical evidence indicating that developing individuals adjust their long-term phenotype according to early environmental demands, we propose that current rearing and housing standards do not adequately prepare experimental subjects to their actual adult environments. Specifically, while the adult life of a laboratory animal is characterized by frequent stimulations and challenges, the neonatal life is dominated by quietness and stability. We suggest that such form of mismatch may remarkably influence the reproducibility and reliability of experimental findings.

## Introduction

“The Snark was a Boojum” [1] is the title of a provocative Presidential address that Prof. Frank Beach delivered before the Division of Experimental Psychology of the American Psychological Association in 1949. The title comes from the quotation of a poem by Lewis Carrol, “The Hunting of the Snark”, the story of ten improbable characters hunting for a prey called “Snark”. Although such prey is generally harmless and tasty, it entails a considerable risk whereby it may be mistaken for its dangerous conspecific “Boojum”. While the one who meets a Snark comes home safe, the one who encounters a Boojum suddenly disappears. Frank Beach paralleled “The Hunting of the Snark” to the direction being taken by comparative psychology in the 1950's and openly criticised the narrow approach that was characterizing this field of investigation back then. He systematically revised a subset of studies (all articles published between 1911 and 1948 in the *Journal of Comparative and Physiological Psychology*) and argued that the term “comparative” was close to lose its original meaning of “involving comparison between two or more branches of science or subjects of study” [2]. His criticism principally grounded upon two specific aspects: (1) paucity of experimental species used in research; (2) limited number of experimental paradigms and domains investigated. With respect to the first aspect, Beach observed that, in spite of the admirable aim to derive fundamental theories resting upon a comparison across multiple species, the majority of experimental studies were conducted on white albino rats. In his analysis, Beach observed that, although the total number of published articles linearly increased between 1911 and 1948, the number of species studied approximately halved during that same time period. More dauntingly, he showed that, towards the end of 1940's, approximately 70% of all published articles were devoted to the Norway rat. With respect to the type of studies conducted, Frank Beach noted that the nature of the experimental paradigms adopted to derive general conclusions was scant. Specifically, he reported that, among the same subset of studies, those devoted to conditioning and learning, reflexes and simple reactions, and sensory capacities accounted for approximately 80% of all studies (with conditioning/learning representing the largest proportion). Other highly relevant fields (e.g. reproductive, social, and emotional behaviours) accounted for a negligible proportion of all studies.

Based on these considerations, Beach equated comparative psychologists with the improbable characters of the poem, and the white albino rats with the alleged “Snark” that ultimately manifested as the “Boojum”. Thus, under the assumption that rats constituted the species of choice in comparative psychology, experimenters employed them with the aim of disclosing the basic processes governing behaviour of a wide spectrum of taxa. With this perspective, rats represented the “Snark” capable of providing scholars with fundamental information about human behaviour. However, such an assumption turned out to be fundamentally wrong, because the conclusions derived from a single experimental species would not generalise to a larger context. Instead of constituting the desired target, laboratory rats turned out to be a limitedly informative tool that, rather than behoving scientists, had the capability to eradicate comparative psychologists. Back in 1950, rather than a “Snark”, laboratory rats epitomized the dangerous “Boojum”.

In this article, we argue that now a days we are facing a similar difficulty in applied research. Specifically, as preclinical neuroscientists, we are hunting a very few experimental “Boojums” (predominantly mice) while being persuaded that they constitute the “Snark” potentially disclosing the mystery of translational research.

## Review

### From non-comparative to non-translational

Although the considerations reported in Beach's article were meant to pertain to comparative psychology, we believe that they are still valid and may generalise to the translational wave that behavioural neuroscientists are currently surfing. The word “translational” in fundamental research has become a sort of dogma that scientists have to adhere to in order to get their research funded and appreciated. Whilst the adjective “translational” originally referred to the process of translating text or words from one language to another, it has recently penetrated the field of biomedical sciences [3], within which it has rapidly attained a pivotal role. A simple PubMed search for the term “translational research” led to 4846 matches with the first reference dating back to 1993 in a study on cancer prevention [4]. Within this realm, “translational” usually refers to the process of gathering evidence collected through different methodologies and transforming them into knowledge advancements (most often treatment/therapies) readily available to patients. Bench-to-bedside constitutes another suggestive phraseology frequently adopted to describe this process [5]. However, in an attempt to reduce high attrition rates [6] during clinical trials and to bridge the gap between preclinical and clinical research, some novel biomedical research approaches also aim at “back-translating” clinical findings to measures in preclinical animal research. The pervasiveness of these concepts is echoed by their presence in names of laboratories (e.g.http://dceg.cancer.gov/about/organization/programs-hgp/ltg; http://hearing-research.med.nyu.edu; https://pharmacyschool.usc.edu/research/core-facilities/translational-lab/; [7]) grant programs (e.g. http://www.cc.nih.gov/ccc/btb/), funded research projects (e.g. EUHFAUTISM: European High-functioning Autism network: Translational research in a phenotypically well characterised sample) and journal articles reporting outcomes of preclinical research (e.g. [8-10]). However, despite the relatively recent use of these phraseologies, translational research has been conducted for many years [11] with the core idea being very close to the traditional - perhaps more general - concepts of “external validity”, i.e. “the possibility to extrapolate the findings obtained within a given experimental context (e.g., strain, species, laboratory, and time of the year) to other situations” [12], and “predictive validity”, i.e. the possibility to predict the efficacy of a therapeutic intervention in patients using animal models [10] (for comprehensive work on validity criteria, please refer to [13,14]). Regardless of the terminology, these considerations reflect the importance that biomedical sciences are giving to the possibility to inform treatment and therapy resting upon information derived from fundamental research.

The attempt to translate fundamental research findings from the laboratory to the human patient entails the acknowledgement of the fact that the steps between the collection of preclinical experimental data and its practical adoption in human-centred functions (*in vivo* or *in vitro*) are arduous and enormous. The widespread failure to translate preclinical animal research to clinical trials [15,16], however, suggests that this is not always taken seriously. In a 10-year review (1991-2000) of drug development, Kola and Landis [15] reported that the success rate from first-in-man to registration for all therapeutic areas was on average 11%, indicating that only one in nine compounds made it through development and finally got approved by the European and/or the US regulatory authorities. And the success rate was even worse for trials in specific research areas, such as oncology or woman's health [15]. Furthermore, a recent finding in the field of inflammation has caused something of an uproar among scientific journalists: out of about 150 potential treatments for severe inflammation that have been found to work in mice not a single one worked in humans [17]. Besides possible shortcomings in the clinical trials that may account for these high attrition rates, findings may also be explained by inadequate and biased animal data, overoptimistic conclusions drawn from methodologically flawed animal studies, and/or by the lack of external validity of some animal models [16]. Thus, before claiming that a given phenomenon pertains to humans, based on preclinical studies, several conditions need to be met. Among these, it should be demonstrated that the phenomenon under investigation extrapolates to other conditions, thereby resisting the challenge of independent testing. Thus, a one dimensional experimental approach, not integrated with converging methodologies, will not allow generalising conclusions to a larger population, let alone to a different species. The proposed *bench-to-bedside* translation shall rest upon studies involving diverse experimental approaches, wherein diversity should encompass theoretical considerations, experimental species under investigation, laboratories, and experimental test paradigms.

### Variability in the animal kingdom

Living organisms do vary; this is a central fact in biology. And besides differing from conspecifics (inter-individual variation), they also display changing behaviour and physiology throughout ontogeny (intra-individual variation). Several scientists have pointed to the sources of variation (genes, environment, and their interaction) [18-20] and proposed theoretical frameworks to explain why variation is key to survival and reproduction [21]. Briefly, individual genomes are the result of evolutionary forces and provide the organism with a set of phenotypes that can vary slightly depending on the specific environmental context (“reaction norms” [22]). Epigenetic programming, the set of molecular mechanisms capable of modulating gene expression -and ultimately individual phenotype - has been proposed as one of the key factors allowing the cross-talk between the environment and gene-expression [20,23]. A large body of clinical and preclinical evidence indicates that precocious environmental influences persistently modulate the individual phenotype [24-27]. However, rather than being a passive receiver of external stimuli, the organism has been proposed to constitute a filter capable of unconsciously exploiting precocious cues as predictors of their future environment. Such predictors, in turn, have been proposed to adjust the individual phenotype in accordance with the actual cues present in the adult environment. Thus, developmental plasticity has been framed within the context of “phenotypic programming”, a hypothesis posing that growing individuals “use” early environmental cues as predictors of their future habitat and accordingly “adjust” their phenotype. This hypothesis can be illustrated with the example of *Daphnia*, a freshwater crustacean that may (or may not) present with a protective “helmet” [28]. While the possibility to develop the helmet depends on specific genes common to all individuals, its actual patterning rests upon epigenetic mechanisms. Specifically, the helmet occurs if developing individuals are exposed to a predator odour, and does not occur in a predator-free environment. Compared to an unprotected *Daphnia*, a helmet-protected individual would generally prevail in the presence of predators but succumb in their absence. The disadvantages encountered by helmet-protected *Daphnia *in a predator-free environment are related to the energetic costs associated with helmet production. Ultimately, long-term survival and reproductive success depend both on precocious cues (presence/absence of predator odour) and on the environment encountered during later stages of life (presence/absence of predators). Matching precocious developments with adult life conditions should promote survival and reproduction, whereas mismatching scenarios should result in poor outcomes in terms of individual fitness [21,28]. Whilst this example pertains to crustaceans, adaptive adjustments to environmental cues have also been ascribed to mammals (e.g. guinea pigs [29,30]) including humans [31-33]. For example, Sachser demonstrated that the neonatal social context permanently adjusts the adult individual response to unfamiliar conspecifics in guinea pigs [29,30]. He showed that adult male guinea pigs, which experienced a complex social environment early in ontogeny (large colonies composed of mixed-sex conspecifics), adapt faster to a group of unfamiliar subjects compared to individuals reared with a single female [29,30]. The thrifty phenotype hypothesis extends to human beings the possibility that precocious cues adaptively calibrate long-term adjustments [31-33]. This hypothesis proposes that nutritional status during the early stages of ontogeny regulate individual metabolism with respect to the ability of accumulating and dissipating energies (i.e. glucose-insulin metabolism) [32]. Specifically, it is suggested that a poor nutritional status *in utero* forecasts adverse adult foraging conditions, which are matched through the patterning of a system capable of accumulating and storing the scant resources available (glucose tolerance) and limiting energy expenditure (e.g. insulin resistance). Such a phenotype would be advantageous under highly challenging foraging conditions but disadvantageous in an environment in which food resources are abundant. Epidemiological studies conducted in a cohort of Dutch individuals born to mothers experiencing severe food shortage during the late stages of pregnancy(due to an embargo during the Second World War) support this notion. Specifically, these individuals, who after a difficult gestation matured in a food-rich environment, showed an increased vulnerability towards type 2 diabetes compared to age-matched controls not facing food shortage during gestation [32]. These results have been interpreted as evidence that gestational food-restriction signaled an adult environment characterized by harsh foraging conditions, and that the individual phenotype adjusted accordingly. Maladaptive adjustments occurred due to the fact that the early environment failed to precisely forecast the adult environment (“phenotypic mismatch” [21]). A resulting thrifty phenotype would have been adaptive in a situation in which food resources were scant. These studies further support the notion that variation in living organisms is a force capable of permitting individual fine-tuning to a changing environment and thereby contributing to survival and reproduction.

Ultimately, these examples indicate that variation is a norm and that it may exert a pivotal evolutionary-adaptive function. Importantly, the experimental evidence in support of this fundamental evolutionary adaptive mechanism stems from converging studies of three different species (neither rats nor mice), and adopting completely different methodologies. By the same token, since these mechanisms generalize to a wide range of tax a, they should also pertain to laboratory rodents. Thus, should neonatal rearing conditions provide laboratory rodents with information about their future habitat, it is tenable to question the extent to which our husbandry strategies favour adaptive plasticity to the lab specific challenges encountered by experimental rats and mice.

### Are laboratory rodents adapted to their living and testing conditions?

In the previous paragraph, we described experimental evidence indicating that environmental cues encountered during the early stages of development adaptively calibrate individual adjustments to adult life conditions [21,24,27]. We also reported evidence indicating that situations in which neonatal pretences do not match adult life conditions may favour vulnerability to pathology [31,32]. Since rats and mice are daily used as models for human function and dysfunction, we may wonder whether their neonatal life conditions match the challenges encountered in adulthood (thereby favouring adaptive plasticity) or whether they are not adequate predictors thereby hampering “normal” development. The consequence of this question is: can control rats and mice be regarded as “normal” individuals? The fact that neonatal life conditions in laboratory facilities do not adequately reflect adult challenges has already been discussed in details [34]. Such proposition rested upon the observation that, while neonatal conditions are pervaded by safety, stability and quietness, adult challenges to a laboratory rodent can occur frequently and in variable ways. For example, single housing, re-grouping, injections, food shortage/deprivation, and cage tilting constitute a subset of stressors present in the daily life of a laboratory individual. Thus, the precocious quietness has been proposed to conflict with the adult life encountered by experimental subjects [34]. To evaluate whether plastic adjustments to neonatal rearing conditions affected the quality of experimental data, we devised a strategy to address the extent to which laboratory animals approximate a “normal” population [35], wherein “normality” has to be considered in statistical terms (Gaussian distribution). Previous studies proposed that while physiological variables should distribute normally in a natural population, the elevated occurrence of abnormal behaviours (due to captivity) may skew data distribution [36]. Resting upon these propositions, we first addressed the data distribution of a large set of data collected in control mice, and then evaluated whether exposing neonate subjects to physiological stressors may favour the exhibition of normal behaviour [35]. The rationale behind this study was the following: (i) laboratory animals are reared under highly stable and safe conditions; (ii) adult life conditions do not match neonatal expectations; (iii) early/adult mismatch hampers adaptive adjustments thereby increasing the rate of abnormal phenotypes and skewing the normality of data distribution; (iv) reducing the early/adult mismatch may reduce the number of abnormal phenotypes and normalize data distribution. In accordance with these predictions, we observed that control data failed to distribute normally and that access to neonatal stressors “normalized” the situation [35]. Thus, although our approach is unlikely to be used on a large scale, it highlights the possibility that laboratory controls do not represent a natural population of individuals (let alone the possibility that they represent a natural population of a different species). However, this study corroborates independent evidence indicating that alternative strategies may increase the translational value of preclinical experiments [6].

Ultimately, the ideas described insofar emphasize the fact that adaptive plasticity and individual variability constitute the norm rather than an exceptional event in the lab. An integrated multifactorial approach aimed at deriving fundamental theories [37] should not disregard this aspect. However, how do we, as behavioural neuroscientists, take account of variability while devising preclinical experimental strategies aimed at gathering information about humans?

### Variability in experimental studies

In spite of its functional significance and widespread acknowledgement of its existence, variability tends to be actively combated by behavioural neuroscientists. Such opposition is openly declared in methodology textbooks that generally propose the adoption of standardised procedures with the aim of increasing “the reproducibility of group mean results from one experiment to another […]” [38]. To achieve this goal, behavioural neuroscientists are recommended to first describe, and then thoroughly standardize, experimental conditions: such homogenisation is expected to range from experimental species and strains, to procedures and contextual factors (e.g. temperature, humidity, and light/dark conditions). These procedures are predicted to render experimental subjects less variable within each study population [39] and, in turn, to increase test sensitivity [39-41]. These theoretical assumptions have led, over the years, to the design of a battery of standardized experimental approaches to which scholars generally adhered. Thus, natural variability has been actively combated via the development of a series of procedures aimed at eradicating variation in core aspects of preclinical research. Specifically, variability has been thwarted at the level of genetics (through the use of inbred strains), rearing and housing environment (through the design of standard laboratory cages), and testing procedures, paradigms, and conditions (see paragraph “What's wrong with “What's wrong with my mouse?””). Although this approach has contributed to remarkable advancements in knowledge, it has also repeatedly demonstrated its own pitfalls. For example, the need to use genetically identical individuals resulted, as of year 2000, in approximately 400 and 200 mouse and rat inbred strains, respectively [42]. Identical individuals have been generated under the assumption that they would generate experimental data characterized by reduced variability [43]. This assumption has been invalidated by many studies over the years. For example, a pioneering experiment performed in 1954 [44] revealed that F1 hybrids performed more consistently in response to pentobarbital than their two parental inbred mouse strains. Converging data, indicating that inbred strains do not necessarily yield consistent results, have been also collected by Crabbe and collaborators ([45], see below for a discussion).

Under the assumption that not only genes, but also the environment contributes to inter-individual variation, behavioural neuroscientists are generally advised to homogenise contextual variables to guarantee within- and between-laboratory experimental reproducibility. The need to standardise housing and rearing conditions stems from abundant literature indicating that a plethora of environmental features may remarkably influence individual phenotype. Whilst macroscopic aspects like environmental enrichment are traditionally considered capable of altering individual physiology and behaviour [46,47], other aspects are not often considered as crucial modulators of individual phenotype. Yet, many intervening contextual variables are capable of skewing experimental results [48]. To give a few examples, the following variables have been shown to alter individual physiology and behaviour [49-52]: ambient noise [53], amount of experimenter intervention during the early stages of life [54-56], levels of maternal care [26,57], location of the cage within the rack [58], and even the gender of the experimenter [59]. Within this scenario, it seems reasonable to ask the following: (i) Is it tenable to propose a homogenisation of all these variables? (ii) If it were possible, would such standardisation really guarantee reproducibility of experimental findings? (iii) And if data were reproducible, does this automatically improve their translational value? Below, we attempt to provide a personal perspective on these questions.

*i. Is it tenable to propose a homogenisation of all these variables?* We believe that this is not attainable, and our concern resides in the fact that some factors that have been shown to modulate the individual phenotype cannot be standardized across different laboratories. Such hard-to-standardise contextual variables are, for example, constituted by position of cages in the rack, daily routines, lighting conditions, humidity, the room architecture, and/or training and individuality of lab personnel [48]. Furthermore, while standardising ambient noise is theoretically attainable, guaranteeing identical shipping conditions is impossible, at least owing to the fact that not all laboratories are equally distant from the commercial breeder (i.e. shipping duration may vary greatly across labs). Thus, as each of the aforementioned variables has been shown to modulate individual phenotype, a full standardisation seems to be theoretically impossible [37,60,61].

*ii. If it were possible, would such standardisation really guarantee reproducibility of experimental findings?* Again, we propose a negative answer to this question. This is related to empirical studies appropriately designed to evaluate the likelihood that identical experimental conditions would yield identical results. For example, despite extraordinary efforts to standardize husbandry and test conditions across three laboratories, Crabbe and colleagues [45] found that some behavioural strain differences were poorly reproducible across sites. Specifically, the authors demonstrated that while some parameters (general locomotion and preference for palatable solutions) yielded consistent and fully reproducible results, other variables (e.g. anxiety-related profiles) were characterised by remarkable inconsistencies in terms of stain x laboratory interactions. Thus, even the direction of strain differences were found to vary between different laboratories. Meanwhile, similar results have been found in several other multi-laboratory studies [62-64], showing that even highly standardized experiments may lead to conflicting test outcomes. Available evidence therefore suggests that although standardisation may favour comparability of data, it cannot fully guarantee reproducibility of experimental findings [64-67]. The study by Crabbe and colleagues [45] further strengthened the view that genetic and environmental homogeneity are not equivalent to data reproducibility (neither within- nor between-laboratories) and that we are not far from what Henderson had already summarised in 1970: “*For the time being, investigators must be aware of the possibilities that early environmental interactions with genotype may limit the validity of their findings to their own unique laboratory situations* (Henderson, 1970, P.509)” [68].

*iii. If data were reproducible, does this automatically improve the translational value?* The third and last doubt concerns the real utility of obtaining fully reproducible results through genetic and environmental homogenisation. As it has been argued before, not all statistically significant effects are necessarily biologically meaningful [69]: perfect and, thus, fully effective standardisation would decrease inter-individual variation to zero, resulting in almost identical individuals within study populations. At the same time, however, the experiment would turn into a single-case study with a sample size of N=1 [70,71], producing statistically significant, but probably irrelevant results. We reckon that data obtained in a single individual would not easily generalise to a larger population of a different species. Furthermore, such a scenario deviates from the original goal to translate experimental findings obtained in laboratory rodents to human beings.

Thus, attempting to standardise experimental conditions to obtain reproducible results seems impractical, ineffective, and - from our perspective - fundamentally useless. Conversely, we may aim at devising experimental test strategies in which variation constitutes the norm (e.g. using different experimental strains and species reared under different conditions to test a given hypothesis) and evaluate whether the mechanism under analysis resists the challenge of testing under variable conditions. Briefly, we believe that translational research should carefully reconsider the fundamental principles of comparative studies, namely (1) the use of more than one experimental species and (2) more than one experimental approach.

### (1) Experimental species: data on animal use across Europe suggest that the mouse is our current “Boojum”

**Figure 1:**
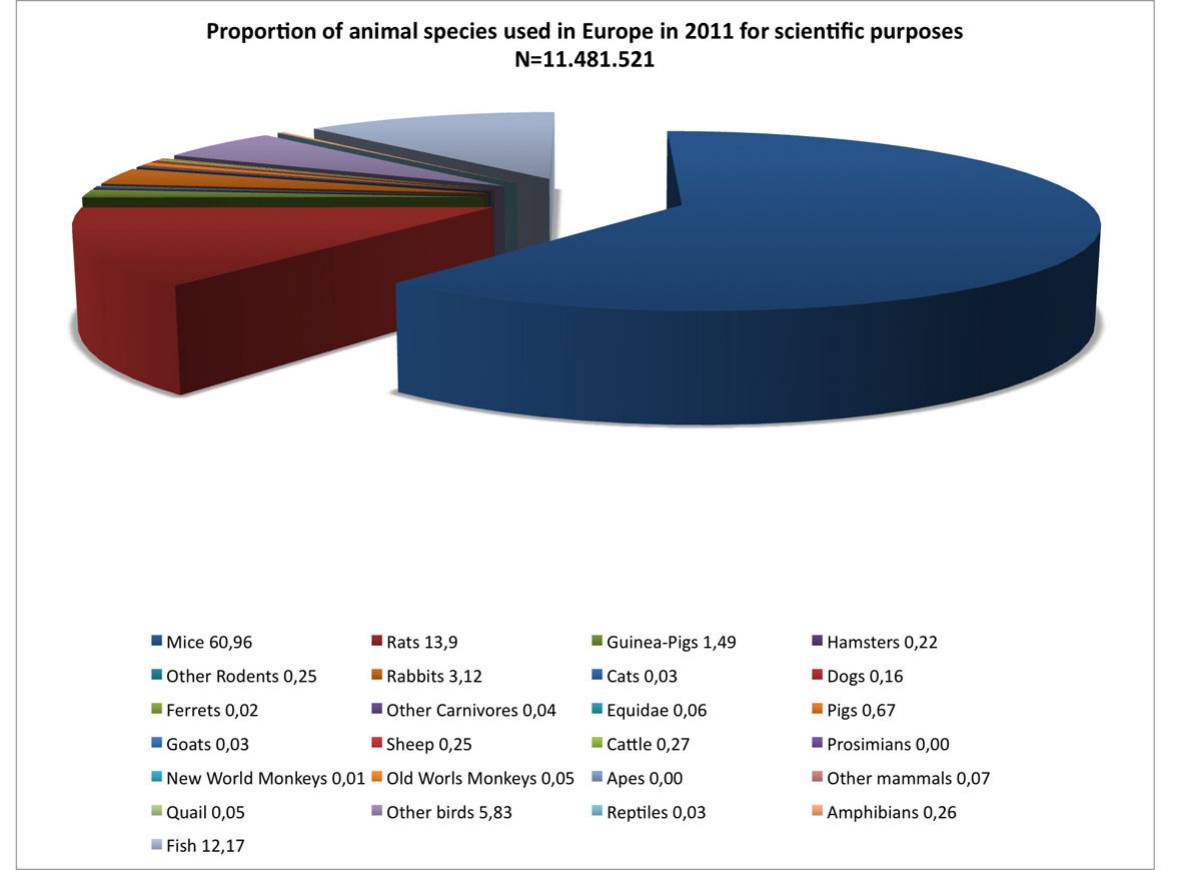
Animal species used with scientific purposes in the European Union in 2011. Per cent values represent the proportion of a given species over the total number of subjects used. Modified from [102].

**Figure 2:**
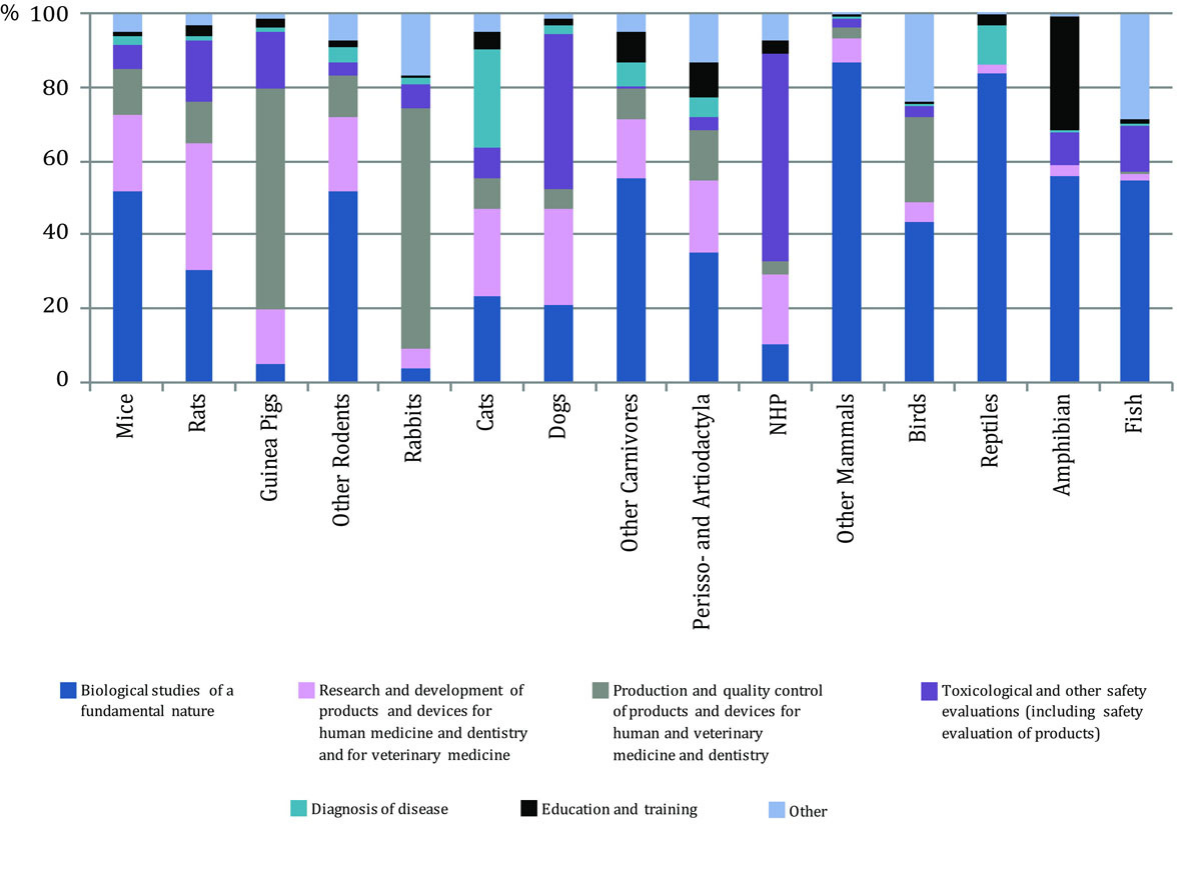
Purposes with which experimental subjects are used in the European Union split by tax a. Modified from [102].

In 1950, Frank Beach was concerned with the fact that white albino rats constituted the majority of animals employed in comparative studies. To support this claim, he restricted his analysis to the research manuscripts published in the *Journal of Comparative and Physiological Psychology* between 1911 and 1948. In order to evaluate how the current status of preclinical research compares to the trend described in 1950, we reviewed information regarding how a large group of scientists utilise animal species for experimental purposes. To this aim, we selected a document in which data concerning the diversity of experimental species (see Figure 1), together with the specific purposes for which they are used in Europe, are systematically described. Such data are available in the “Report from the commission to the council and the European Parliament: Seventh Report on the Statistics on the Number of Animals used for Experimental and other Scientific Purposes in the Member States of the European Union” (http://eur-lex.europa.eu/legal-content/EN/TXT/?uri=CELEX:52013DC0859). This analysis is certainly limited as it does not include invertebrates, and only pertains to data collected in Europe. Yet, it reports data on 11.481.521 animals used with scientific and experimental purposes in 2011 (Biological studies of fundamental nature, 46.1%; Research and development of products and devices for human medicine and dentistry and for veterinary medicine, 18.8%; Production and quality control of products and devices for human and veterinary medicine and dentistry, 13.9%; Toxicological and other safety evaluations (including safety evaluation of products), 8.8%; Diagnosis and disease, 1.6%; Education and training, 1.6%; Other, 9.3%). As reported in Figure 1, mice occupy the largest proportion of all animals used with all the aforementioned purposes. Although many species are listed, it appears very clear that a minority of them constitute the vast majority of all animals used. Thus, mice, rats, and fish (the entire class not otherwise specified) account for approximately 87% of all experimental animals used. The report also describes how animals have been used with respect to the specific field of investigation (see Figure 2). Several aspects may explain the reason why these experimental species have become more popular over the years. While some of them, like genetic proximity between humans and the model species [72,73], have a solid theoretical grounding, some others, like increased stocking density, fast intergeneration time [74], and availability of standardised experimental tools, appear more related to practical and economic considerations than scientific evidence. To obtain information regarding the diversity of experimental species used in preclinical research, the analysis was limited to the “Biological studies of a fundamental nature” which account for 46.1% of all animals used in Europe. To this aim, the information of Figure 1 and Figure 2 was scaled for the 46.1% value. This approach indicates that the role exerted by these three tax a in preclinical research is even more prominent. Thus, in the year 2011, of all animals used in biological studies of a fundamental nature, approximately 70% were mice, 13% fish, and 8% rats. All the other species accounted for the remaining 9%. Thus, notwithstanding the limits of this analysis, it seems tenable to propose that the number of animal species currently adopted in preclinical research may reflect a form of an overspecialisation rather than a generalization and may thus not possess an adequate translational value.

### (2) Experimental approach: what's wrong with “What's wrong with my mouse?”?

As mentioned in the Introduction, beside the limited number of species investigated, another critical aspect in Comparative studies was associated with the paucity of experimental test paradigms adopted. A pivotal concern emphasised by Frank Beach was that, notwithstanding the goals, most of comparative psychology research boiled down to conditioning experiments performed in Skinner boxes. While this aspect is no longer as critical as in 1950 - many different tools are currently available to behavioural neuroscientists - we argue that much should be done in order to guarantee that a given domain be analysed from converging perspectives and not addressed adopting a single approach. Currently, despite the presence of many different strategies, the homogenisation effort attempts to constrain the number of validated “standard procedures” to be used while investigating a given domain [75]. Although this limitation varies across different experimental domains, fields like toxicology and biomedical research often rest upon a limited array of ‘standard’ tests. The latter are generally constituted by a set of specific end points, such as sensory-motor function, anxiety/depression-like behaviours, general health, general metabolism, and cognition [76-79]. The idea behind these efforts is to set a common ground for all behavioural neuroscientists interested in a given domain and provide them with rigorously standardised operating procedures. An extreme example of this approach is constituted by SHIRPA (the acronym of “Smith Kline Beecham, Harwell, Imperial College, Royal London Hospital, phenotype assessment” [80]), a set of common procedures aimed at aiding behavioural biologists in the evaluation of the “aberrant biology seen in mouse mutations and identification of more subtle phenotype variation” [80]. SHIRPA is a clearly defined test protocol entailing three stages, each devoted to a specific domain: the first stage consists of an observational screen not requiring the use of specific tests; the second stage consists in the administration of classical tests evaluating e.g. locomotion, anxiety-related behaviours, and motor coordination; finally, the third stage involves the adoption of more complex experimental paradigms evaluating complex cognitive abilities (learning and memory), startle reactivity, and sensory-motor gating (pre-pulse inhibition) [81,82]. In a different approach, a large-scale three-year European initiative launched in 2002 involved 17 laboratories with the aim of disseminating “a large number of primary screen ‘Standard Operating Procedures’ [and] ensure that results coming from labs adhering to the protocol are comparable”. The rationale for this initiative was that “many labs use their own particular research protocols for screening mice, making it difficult to accurately compare and share data from different sources” (http://ec.europa.eu/research/health/genomics/projects/eumorphia_en.htm). Thus, in spite of considering variability as natural, and attempting to reconcile inter-laboratory differences within larger theories, the approach of an influential portion of the scientific community is to eradicate it through SOPs. This attitude is further exemplified by “What's wrong with my mouse?” [83], a bestselling textbook devoted to: behavioural neuroscientists delving into the field of genetically engineered mice; and geneticists attempting to phenotype their disease models. Using the author's words, this book “is written for these pioneering molecular geneticists, and for the talented students who will be the next driving force in moving the field forward” [83]. This detailed book contains a comprehensive list of finely described screening tasks that may be used while investigating a plethora of domains. The author, Jacqueline Crawley, clearly states that this text should constitute a reference book for those approaching the field of behavioural neuroscience, but that the analysis should not be limited to the protocols described therein. While having access to such a text certainly constitutes an advantage for the field, scientists should be aware of the fact that a differential approach is needed in order to attain externally valid data. To briefly answer the provocative question of the paragraph title, we suspect that nothing is wrong with “What's wrong with my mouse?”, as long as it constitutes the starting and not the end point of preclinical research.

An additional concern directly linked to such paucity in experimental test paradigms is the fact that they are often translated from one species to another (e.g. from rats to mice), from one strain to another (e.g. from outbred to inbred mice) or from one sex to another without a preliminary consideration of the eco-ethological value of the tests themselves. Examples in which such translation is open to criticisms are abundant. For example, one of the most widely used paradigms to investigate memory in rodents is constituted by the Morris water maze task [84]: this task was originally developed “to demonstrate that rats can rapidly learn to locate an object that they can never see, hear, or smell provided it remains in a fixed spatial location relative to distal room cues” [84]. In this test, rats are required to swim in order to locate a platform, hidden beneath the water surface, resting upon environmental cues. This task which, over the years, yielded fundamental evidence regarding the biological mechanisms underlying learning and memory [85], rests upon rats’ natural swimming capabilities. In contrast with rats, mice do not show analogous swimming skills thereby not being particularly suited to this test. Notwithstanding the difference in preparedness towards swimming, and despite the availability of dry versions of this task [86,87], the water maze task is still currently used in countless mouse experiments (a simple Medline search with the search terms “Morris maze mouse”, limited to the year 2014, retrieved 330 manuscripts). Analogous concerns can be raised as to several operant-based tasks directly translated from humans to rodents. The attentional set-shifting task constitutes one such example. This task evaluates the cognitive flexibility of an individual by addressing its capability to disregard an acquired rule in favour of a novel one. In humans, this capability is addressed through the Wisconsin Card Sorting task [88]: subjects have to learn to discriminate between two cards - differing along two or more dimensions (e.g. colour and number) - using one dimension (e.g. always red regardless of the number). Once the rule is acquired, subjects are required to disregard it in lieu of a different one. Shifting can be intra-dimensional (e.g. from one colour to another) or extra-dimensional (e.g. from colour to number). The original rodent versions of this task were based on operant-cage testing and required weeks of training to observe consistent non-match-to-sample rule learning [89]. Extensive training was due to the fact that the ethological relevance of this task was near-to-nothing for the experimental subjects. Later incarnations of this test kept into account the fundamental needs and abilities of laboratory rodents by providing them with digging bowls (rather than operant screens) varying across three ethologically-relevant dimensions: odour, texture and digging medium [90,91]. This simple methodological variation reduced learning curves from three weeks to approximately 100 trials [90,92-94]. The importance of taking individual fundamental needs into account also extends to social and defensive behaviours. Although there are marked similarities in the social organization and behaviour of mice and rats - both have territorial and colonial social systems where one male maintains a territory containing one or more reproductive females - there exist some striking differences. For example, with respect to social play juvenile rats engage in much more play fighting than mice do. Furthermore, rats and mice have been described to differ widely in conspecific aggression, food defence and predatory defence [95], challenging the common practice to simply translate social tests, such as the resident intruder test or the social interaction test, from rats to mice or *vice versa*. However, as it is not possible to evaluate the ethological validity of each common test used in rodent studies at this point, we would like to highlight again that far too often experimental paradigms are translated from one situation to another or from one species to another without considering individual needs and natural predispositions (for a detailed description of the importance of ethologically relevant experimental paradigms, the interested reader is referred to the seminal work of Bredland and Bredland [96] and Dewsbury [97]). Thus, what might be an easy, readily accomplished task for one species (or individual) may be less fitted or even beyond the evolved repertoire of another species, further supporting the need for a paradigm shift in animal experimentation.

## Conclusions

We are certainly not the first to re-evaluate the seminal considerations proposed by professor Frank Beach in 1950 [98,99]. Together with other colleagues, we share the view that our current approach in preclinical animal research is too narrow both in terms of experimental species investigated and experimental test paradigms adopted to address specific domains (e.g. [10,65,75]). Compared to the situation of 65 years ago, however, we are now fully aware of the fact that different methodologies exist and that over-specialisation and standardisation will not allow an easy translation of preclinical findings. To improve the situation, some first heterogenisation strategies have been proposed [64,66,67]. In these studies, [66] strain differences in several behavioural domains were investigated on the basis of experiments in heterogeneous experimental populations. Briefly, to test whether highly standardised, i.e. homogenised, experiments yielded more consistent results than heterogenised experiments, we compared data obtained in a population of individuals reared under a given standardised condition with data obtained in a population of individuals reared in different environmental conditions. The results demonstrated that heterogenised experiments yielded more stable results across experiments and were characterised by a reduced number of false positive results compared to homogenised experiments [66]. These findings support the view that alternative experimental strategies may indeed enhance the reproducibility and translational value of preclinical animal research. However, although these efforts were limited to housing and testing conditions, we believe that this approach may also apply to heterogenisation of both experimental species and testing paradigms.

Thus, available literature indicates that re-introducing natural variation in translational research may favour the collection of data potentially informing human-centred innovative knowledge and therapeutic approaches. Although a complete description of an innovative experimental paradigm is beyond the scopes of the present manuscript, we will briefly outline some factors to be considered before a heterogenised approach can be implemented into practice:

1) Increasing the number of species analysed: as described above, three species account for approximately 91% of all fundamental research studies conducted in vertebrates. Incorporating additional experimental species seems to constitute a fundamental goal.

A potential limitation to this approach relates to the difficulty of establishing all the facilities required to perform experiments on non-standard animals. Yet, data reported in Figure 1 indicate that, at least in the European Union, laboratories and facilities have access to more than 20 different experimental species. Thus, rather than aiming at extending the number of available experimental species, future efforts may aim at taking advantage of existing forces and balancing the use of different species. With respect to economic counter-arguments, we believe that devising large projects in which a given phenomenon is directly tackled through a comparative approach is feasible and does not require large investments to establish novel infrastructures.

Although the economic burden of this transition may be overcome at limited costs, the potential ethical implications may require a systematic consideration. The ethical costs may encompass both the number of subjects to be used and the level of affection towards the species under investigation. With respect to the former, we note that the inclusion of systematic variation does not necessarily inflate the number of subjects to be used in a single study. Combined with targeted experimental designs (e.g. matched-pairs, split-plot, factorial, or randomized block designs) and adequate analytical techniques (e.g. matching, blocking, or stratification), such experimental heterogenisation may be implemented in systematic and controlled ways without the need for larger sample sizes. In particular, the use of randomized block designs has been discussed as being promising in this context [61]. Originally derived from agricultural research, randomized block designs are used to split an experiment into a number of “mini-experiments” according to the natural structure of the experimental material [100]. With respect to animal experimentation, this may lead to a grouping (“blocking”) of experimental subjects on the basis of certain characteristics (e.g. age, prenatal or postnatal experiences, housing conditions, etc.) so that the animals are as homogeneous as possible within blocks, but different between blocks. Because between-block variation can then be eliminated by comparing treatments within blocks only, statistical power and precision are much higher than in a comparable unblocked design. With respect to the latter, we are aware of the fact that the proposition of a larger battery of experimental species may entail the adoption of animals toward which the general public has an elevated affection (e.g. pets). Yet, theoretical considerations and moral appreciation of value contribute to determine the boundaries within which a given animal species shall be used in experimental studies or not. From a theoretical perspective, the European Directive 2010/63/EU explicitly promotes “the use of species with the lowest capacity to experience pain, suffering, distress or lasting harm that are optimal for extrapolation into target species” [101]. Thus, future studies adopting experimental animals characterized by a central nervous system more complex than rats or mice are, at least in Europe, unlikely. Although this aspect warrants a careful control, heterogeneous experiments may therefore beget some ethical advantages. Furthermore, besides evaluating which individuals are used in each single experiment, a proper ethical evaluation shall entail a systematic cost-benefit analysis. In the Directive 2010/63/EU, the latter constitutes a primary parameter to be considered before providing the authorization to use an animal for scientific purposes. Within this realm, it is plausible to envision a full array of different scenarios: e.g. rodent-centred studies forbidden due to limited utility and pet-centred studies allowed due to exceptional anticipated benefits, or *vice versa*.

An additional hurdle to be overcome while devising heterogenised experiments is constituted by the experimental design and the statistics used to analyse data collected under heterogeneous conditions. We are fully aware of the fact that such an experimental design would depart from traditional approaches. By the same token, several design variants are already available taking into account variation in a systematic way without increasing sample sizes (see above, [64]). Considering statistical approaches, principal component and cluster analyses as well as linear mixed models may be promising analytical tools to account for higher within-study variation.

2) Increasing the number of test paradigms used: incorporating additional experimental approaches and paradigms would greatly benefit the relevance of translational research. Contrary to intuition, methodology has been reduced for reasons of efficiency and long-lasting traditions throughout the last decades. However, new technology and sophisticated analysis methods (e.g. home-cage based systems, touch screens) are now available that may foster the implementation of new and innovative test paradigms in a feasible and manageable way.

While the possibility to introduce heterogeneity systematically in experimental studies is still at its inception, infrastructural and theoretical needs are already available to scientists. To conclude, we would like to use the words of Frank Beach, who inspired this article from the very beginning to the very end: “*This will sometimes mean sacrificing some of the niceties of laboratory research in order to deal with human beings under less artificial conditions. It may also mean expanding the number of non-human species studied and the variety of behavior patterns investigated*” [1].

## Declarations

Publication costs for this article were funded by the German Research Foundation (FOR 1232) and the Open Access Publication Fund of Bielefeld and Muenster University.

## Competing interests

The authors declare that they have no competing interests

## Authors contributions

SM wrote the first draft of the manuscript; HR reviewed the original version, added important information and consolidated its structure. SM and HR reviewed the subsequent elaborations of the manuscript and agreed upon the final version.
